# Using matrix frame to present road traffic injury pattern

**DOI:** 10.1186/s40621-018-0154-y

**Published:** 2018-04-23

**Authors:** Chien-Hsing Wang, Wan-Hua Hsieh, Fu-Wen Liang, Tsung-Hsueh Lu

**Affiliations:** 1 Division of Plastic Surgery, Department of Surgery and Trauma Center, Hualien Tzu Chi Hospital, Buddhist Tzu Chi Medical Foundation, Hualien, Taiwan; 2 School of Medicine, Tzu Chi University, Hualien, Taiwan; 3 Department of Public Health, Tzu Chi University, Hualien, Taiwan; 4 Department of Public Health, College of Health Sciences, Kaohsiung Medical University, Kaohsiung, Taiwan; 5 NCKU Research Center for Health Data and Department of Public Health, National Cheng Kung University, Tainan, Taiwan; 6 Department of Public Health, College of Medicine, National Cheng Kung University, No. 1, Dah Hsueh Road, Tainan, 701 Taiwan

**Keywords:** Transport accidents, Road traffic injuries, Pattern of injury, Epidemiology

## Abstract

**Background:**

Although many epidemiological studies have presented road traffic injuries (RTIs) according to the victim’s mode of transport, very few have mentioned the mode of transport of the victim’s counterparts. We sought to use matrix frame to present the pattern of RTIs based on the International Classification of Diseases, Tenth Revision (ICD-10) codes.

**Methods:**

Patients admitted to Hualien Tzu Chi Hospital, Taiwan, for RTIs from January 1, 2013 to December 31, 2016 were included. The numbers and proportions of various crash types of RTIs were presented using a matrix frame. The row margin of the matrix is the second character of ICD-10 codes V00–V79 (victim’s mode of transport), and the column margin of the matrix is the third character of ICD-10 codes V00–V79 (mode of transport of victim’s counterpart), constituting a 80-cell grid.

**Results:**

In total, 2727 patients were included. The cell with the highest proportion in the matrix grid was ICD-10 code V23 “motorcycle rider injured in collision with car, pick-up truck or van” (27.0%, 737/2727), followed by that of V27 “motorcycle rider injured in collision with fixed or stationary object” (12.5%, 342/2727) and V28 “motorcycle rider injured in noncollision transport accident” (12.2%, 334/2727). The matrix pattern of RTIs differed with sex and age.

**Conclusions:**

By using the matrix frame, we can easily understand the RTI pattern for different demographic groups and identify the priority crash types.

## Background

Although many epidemiological studies have presented road traffic injuries (RTIs) according to the victim’s mode of transport (e.g., pedestrian, bicycle, two-wheel motorcycle, and car) (Chokotho et al. 2013; Majdan et al. 2015; Spoerri et al. 2011; Watson et al. 2015), very few have mentioned the mode of transport of the victim’s counterparts, which could provide a more complete picture of the crash event, thus facilitating the design of relevant intervention programs. For instance, in the Netherlands, bicyclists injured in crashes not involving motor vehicles had a higher number of serious injuries than bicyclists injured in crashes involving motor vehicles; in addition, they had different implications for prevention measures, such as the design of bicycle tracks, mobility advice for older bicyclists, and campaigns to encourage bicycle light use (Weijermars et al. 2016).

Compared with the International Classification of Diseases (ICD), Ninth Revision (ICD-9) codes, an innovative feature of the ICD, Tenth Revision (ICD-10) codes for RTI is the use of the mode of transport modular coding frame (Langley and Chalmers 1999), which can be arrayed as a matrix (National Center for Health Statistics 2013). The row margin of the matrix is the second character of ICD-10 codes V00–V79 (victim’s mode of transport), and the column margin of the matrix is the third character of ICD-10 codes V00–V79 (the mode of transport of victim’s counterpart), constituting a 80-cell grid (Table 1). However, no study thus far has presented the RTI pattern by using a matrix frame. In this study, we sought to use matrix frame to present the RTI pattern among people admitted to one medical center in Eastern Taiwan affected by RTI.

**Table 1:** Mode of transport matrix frame based on International Classification of Diseases, Tenth Revision (ICD-10) codes V00–V79

	Mode of transport of the victim’s counterpart									
The victim’s mode of transport	VX0 pedestrian or animal	VX1 pedal cycle	VX2 two- or three-wheeled motor	VX3 car, pick-up truck or van	VX4 heavy transport vehicle or bus	VX5 railway train or railway vehicle	VX6 other nonmotor vehicle	VX7 fixed or stationary object	VX8 noncollision	VX9 other and unspecified
V0 Pedestrian	V00	V01	V02	V03	V04	V05	V06	–	–	V09
V1 Pedal cyclist	V10	V11	V12	V13	V14	V15	V16	V17	V18	V19
V2 Motorcycle rider	V20	V21	V22	V23	V24	V25	V26	V27	V28	V29
V3 Occupant of three-wheeled motor vehicle	V30	V31	V32	V33	V34	V35	V36	V37	V38	V39
V4Car occupant	V40	V41	V42	V43	V44	V45	V46	V47	V48	V49
V5 Occupant of pick-up truck or van	V50	V51	V52	V53	V54	V55	V56	V57	V58	V59
V6 Occupant of heavy transport vehicle	V60	V61	V62	V63	V64	V65	V66	V67	V68	V69
V7 Bus occupant	V70	V71	V72	V73	V74	V75	V76	V77	V78	V79

## Methods

We included patients admitted to Hualien Tzu Chi Hospital, Taiwan, for RTIs from January 1, 2013 to December 31, 2016 and extracted their demographic data (age and sex), ICD-10 codes of external causes of RTIs (V00–V79). The numbers and proportions of various crash types of RTIs were presented using a matrix frame. The users of the matrix can identify the victim’s mode of transport (e.g., ICD-10 code V2 motorcycle rider) in row margin first and then the mode of transport of counterpart (e.g., ICD-10 code VX3 car) in column margin and get the number and proportion of cases. To properly interpret of the comparisons of proportions between different crash types, we calculated 95% confidence intervals for each proportion.

We used the user-friendly self-service business intelligence software Tableau to create a dashboard; therefore, we could select the dimension (specific age group or sex) of our choice. To more clearly visualize the crash types occurring in high proportions, we used a highlighted table: the darker the cell color, the higher the percentage of a particular crash type was. The number and 95% confidence interval of each proportion are displayed in tooltips that pop out when the user hovers over the mark.

## Results

**Fig. 1:**
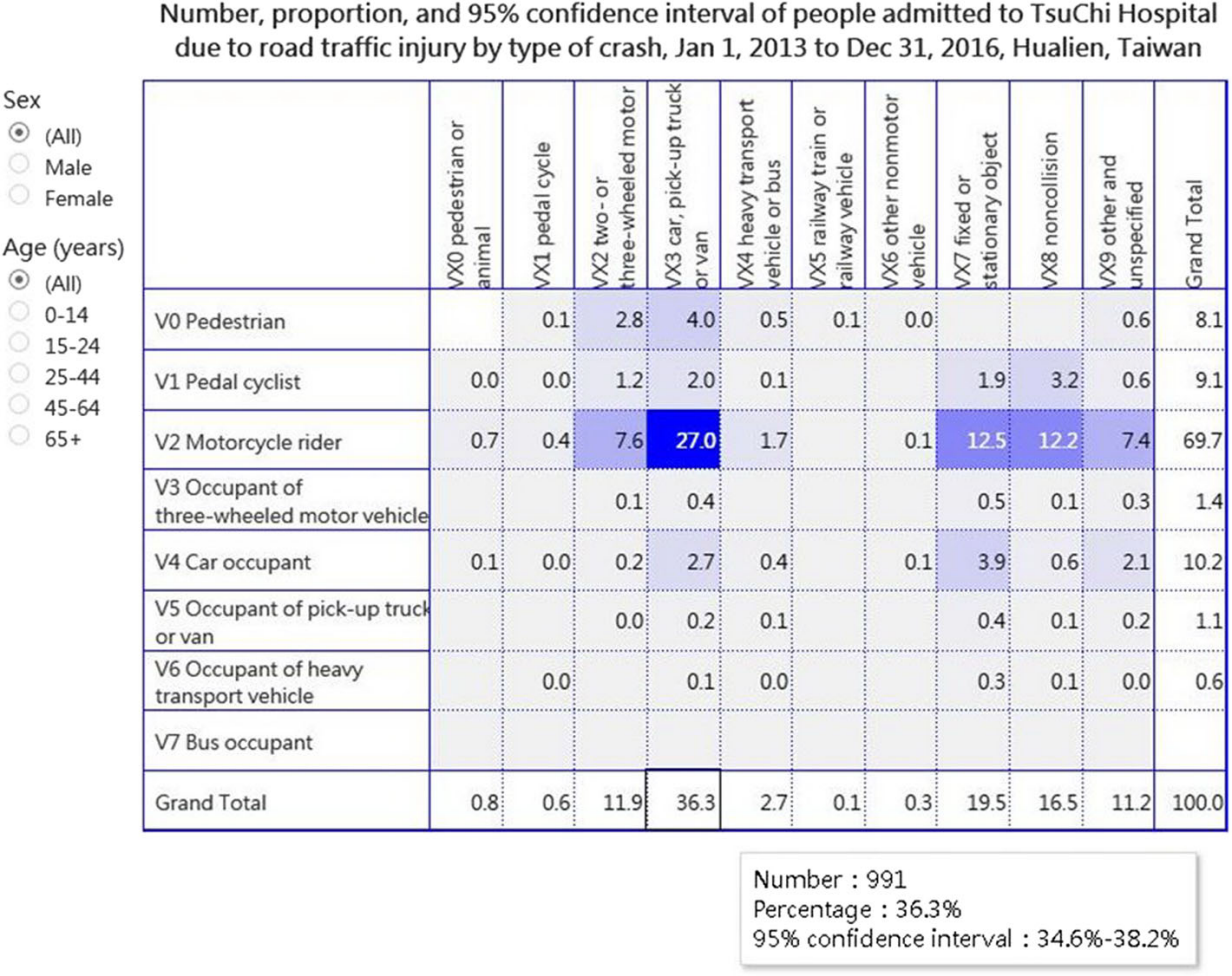
Road traffic injury matrix (https://public.tableau.com/profile/robert.lu#!/vizhome/Matrix_4/Matrix)

A total of 2727 patients were included in the analysis. According to the row margin of the matrix (victim’s mode of transport: second character of ICD-10 codes V00–V79), the ICD-10 code V2 “motorcycle rider” accounted for the highest proportion (70%, 1901/2727; Fig. 1); furthermore, the proportions of V2 for victims aged 0–14, 15–24, 25–44, 45–64, and > = 65 years were 35%, 85%, 71%, 66%, and 65%, respectively.

In the column margin of the matrix (the mode of transport of victim’s counterpart: third character of ICD-10 codes V00–V79), ICD-10 code VX3 “car, pick-up truck or van” accounted for the highest proportion (36%, 906/2516); moreover, the proportions of VX3 for victims aged 0–14, 15–24, 25–44, 45–64, and > = 65 years were 38%, 41%, 32%, 34%, and 38%, respectively.

Of the 80 cells in the matrix grid, the cell with highest proportion (darkest color) in the matrix was that of V23 “motorcycle rider injured in collision with car, pick-up truck or van” (27.0%, 737/2727; Fig. 1), followed by that of V27 “motorcycle rider injured in collision with fixed or stationary object” (12.5%, 342/2727) and V28 “motorcycle rider injured in noncollision transport accident” (12.2%, 334/2727). The proportion of V23 for female patients was 29.5% (359/1216), which is higher than that for male patients (25.0%, 378/1511).

**Fig. 2:**
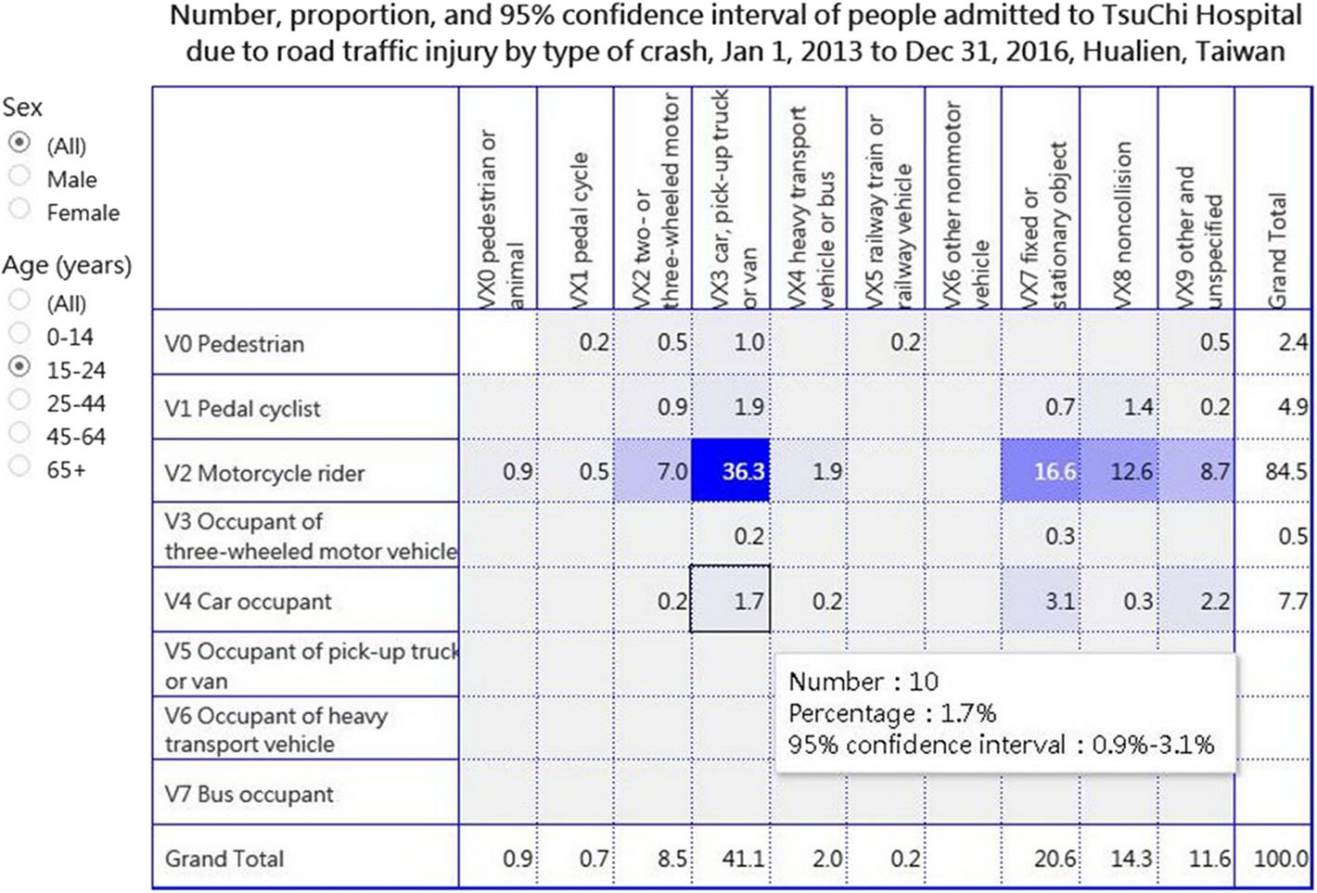
Road traffic injury matrix for patients aged 15–24 years

**Fig. 3:**
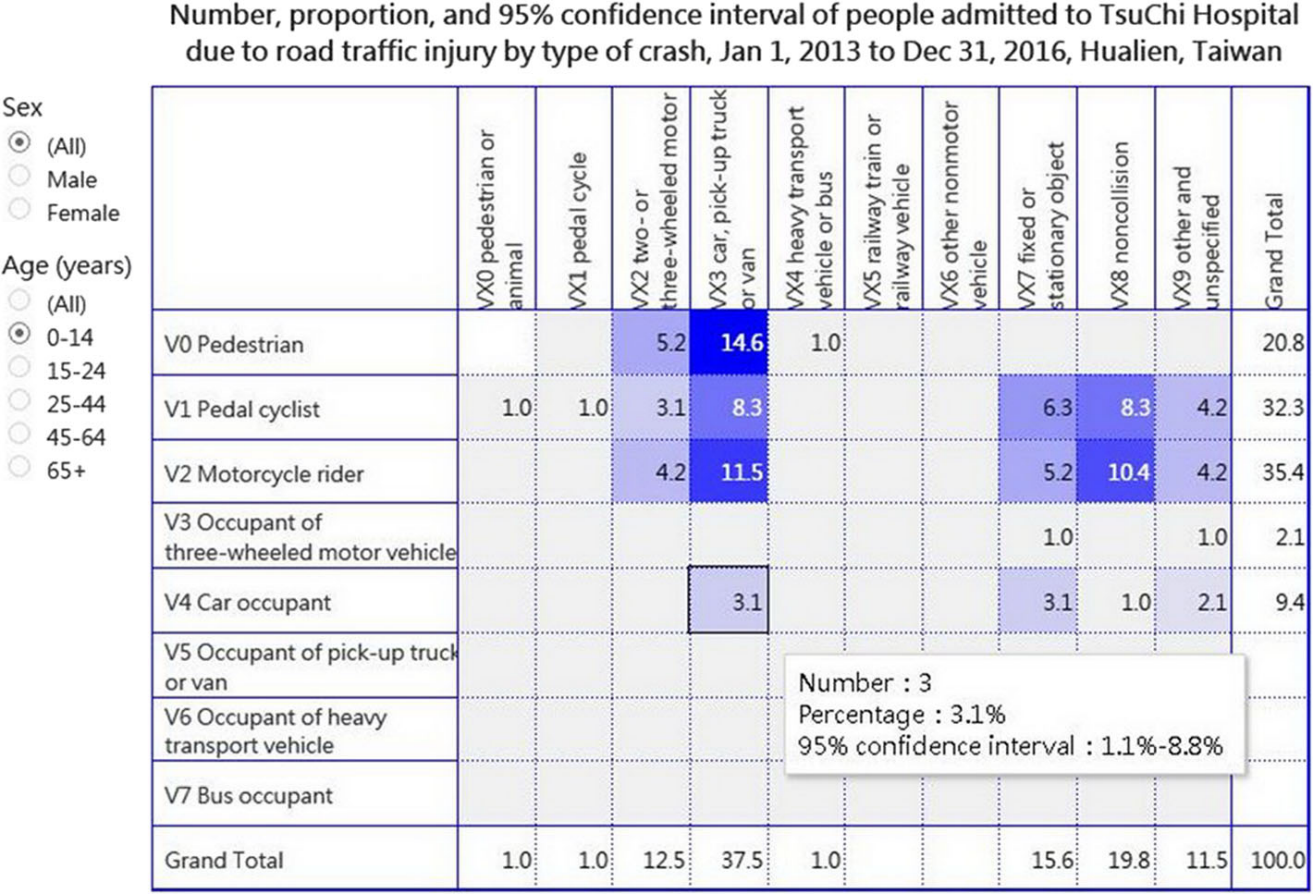
Road traffic injury matrix for patients aged 14 years and younger

For patients aged 15–24 years, the cell with the highest percentage of RTIs was that of V23 (36.3%, 213/586), followed by that of V27 (16.6%, 97/586) and V28 (12.6%, 74/586) (Fig. 2). However, for patients aged 0–14 years, the cell with the highest percentage of RTIs was that of V03 “pedestrian injured in collision with car, pick-up truck or van” (14.6%, 14/96), followed by that of V23 (11.5%, 11/96) and V28 (10.4%, 10/96) (Fig. 3).

## Discussion

The findings of this study demonstrate that the main mode of transport of RTI victims in Taiwan was the motorcycle, accounting for seven-tenths of all RTIs. By contrast, in the Netherlands, bicycles accounted for three-fifths of all RTIs in 2011 (Weijermars et al. 2016). Therefore, the RTI patterns may differ considerably among countries.

Regarding the mode of transport of the victim’s counterpart, the number of injured cyclists in crashes not involving motor vehicles was 5 times the number of injured cyclists in crashes involving motor vehicles in the Netherlands (Weijermars et al. 2016). However, according to our findings, in Taiwan, the number of injured bicyclists in crashes involving motor vehicles (ICD-10 codes V23, V24, and V25) was only 1.5 times the number of injured bicyclists in crashes not involving motor vehicles (ICD-10 codes V20, V21, V26, V27, and V28).

Despite the differences in the RTI pattern between Taiwan and the Netherlands, the proportion of RTIs in vulnerable road users was similar between countries: 88% in Taiwan and 86% in the Netherlands. The term “vulnerable road user” refers to people at the highest risk in traffic; these people are not protected by an outside shield, such as pedestrians, bicyclists, and motorcyclists, and have few or no external protective devices to absorb energy in a collision, making them the weak counterpart in a road traffic crash (Costant and Lagarde 2010). Several measures for preventing RTIs among vulnerable road users (e.g., helmet use, conspicuity aids, and avoiding alcohol use) could be applied to motorcyclists in Taiwan and bicyclists in the Netherlands.

Different matrix frame formats have been proposed for presenting injury-related statistics. The most well-known is the Barell matrix, which details affected body region and nature of injury (e.g., fracture) (Barell et al. 2002; Fingerhut and Warner 2006). Another matrix is the external cause of injury mortality matrix, which details RTIs by the mechanism and intent of injury (McLoughlin et al. 1997; Fingerhut and McLoughlin 2001; Fingerhut 2004; Minino et al. 2006). By using the external cause of injury mortality matrix, we determined that the decrease in the mortality trends of some unintentional injuries might be due to the increase in mortality trends of the same mechanism of injury with an undetermined intent (Lu 2002). However, no study thus far has used the mode of transport matrix frame to present the RTI pattern.

This study has two strengths: (1) the use of the mode of transport matrix frame to present the pattern of RTIs and (2) the use of a visualization dashboard to select the demographic group of choice.

However, this study also has several limitations. First, the mode of transport matrix frame is constructed on the basis of the ICD-10 codes; therefore, if the medical record documentation of the mode of transport is not as specific as required, many RTIs will be classified as “unspecified”; thus, no useful information can be obtained. Nevertheless, the quality of health record documentation on the mode of transport at Hualien Tzu Chi Hospital is relatively high: only 11% of cases have been classified as ICD-10 code VX9 “other and unspecified”. Second, the information of only two dimensions (second and third characters of ICD-10 codes V00–V79) could be presented in the matrix. The information of the fourth character of ICD-10 codes V00–V79 regarding whether the motor vehicle occupant was the driver or passenger and whether it was a traffic or nontraffic accident could not be presented in the same matrix. A solution to this limitation is the use of the drill-down function in the visualization dashboard. Third, because RTIs may be concentrated under particular crash types, the number of RTIs in many cells of the matrix remains zero. In other words, the presentation of the RTI pattern by using a matrix frame may occupy a larger space than that occupied by the traditional presentation method.

## Conclusion

In conclusion, by presenting the mode of transport of both the victim and the victim’s counterpart in a matrix frame, we could easily understand the RTI pattern and identify the priority crash types. Studies using matrix frames to compare RTI patterns between countries with different modes of transport are warranted.
